# An Interdisciplinary Approach to Study the Performance of Second-generation Genetically Modified Crops in Field Trials: A Case Study With Soybean and Wheat Carrying the Sunflower HaHB4 Transcription Factor

**DOI:** 10.3389/fpls.2020.00178

**Published:** 2020-03-06

**Authors:** Fernanda Gabriela González, Nicolás Rigalli, Patricia Vivian Miranda, Martín Romagnoli, Karina Fabiana Ribichich, Federico Trucco, Margarita Portapila, María Elena Otegui, Raquel Lía Chan

**Affiliations:** ^1^ Estación Experimental Pergamino, INTA, CITNOBA, CONICET-UNNOBA, Pergamino, Argentina; ^2^ CIFASIS, Universidad Nacional de Rosario—CONICET, Rosario, Argentina; ^3^ Instituto de Agrobiotecnología Rosario (INDEAR)/BIOCERES, Rosario, Argentina; ^4^ CONICET, Buenos Aires, Argentina; ^5^ Instituto de Agrobiotecnología del Litoral, Universidad Nacional del Litoral—CONICET, Facultad de Bioquímica y Ciencias Biológicas, Santa Fe, Argentina; ^6^ CONICET-INTA-FAUBA, Estación Experimental Pergamino, Facultad de Agronomía Universidad de Buenos Aires, Pergamino, Argentina

**Keywords:** transgenic wheat, transgenic soybean, HaHB4, sunflower transcription factor, drought tolerance, grain yield determination

## Abstract

Research, production, and use of genetically modified (GM) crops have split the world between supporters and opponents. Up to now, this technology has been limited to the control of weeds and pests, whereas the second generation of GM crops is expected to assist farmers in abiotic stress tolerance or improved nutritional features. Aiming to analyze this subject holistically, in this presentation we address an advanced technology for drought-tolerant GM crops, upscaling from molecular details obtained in the laboratory to an extensive network of field trials as well as the impact of the introduction of this innovation into the market. Sunflower has divergent transcription factors, which could be key actors in the drought response orchestrating several signal transduction pathways, generating an improved performance to deal with water deficit. One of such factors, HaHB4, belongs to the homeodomain-leucine zipper family and was first introduced in Arabidopsis. Transformed plants had improved tolerance to water deficits, through the inhibition of ethylene sensitivity and not by stomata closure. Wheat and soybean plants expressing the *HaHB4* gene were obtained and cropped across a wide range of growing conditions exhibiting enhanced adaptation to drought-prone environments, the most important constraint affecting crop yield worldwide. The performance of wheat and soybean, however, differed slightly across mentioned environments; whereas the improved behavior of GM wheat respect to controls was less dependent on the temperature regime (cool or warm), differences between GM and wild-type soybeans were remarkably larger in warmer compared to cooler conditions. In both species, these GM crops are good candidates to become market products in the near future. In anticipation of consumers’ and other stakeholders’ interest, spectral analyses of field crops have been conducted to differentiate these GM crops from wild type and commercial cultivars. In this paper, the potential impact of the release of such market products is discussed, considering the perspectives of different stakeholders.

## Introduction

The challenge imposed by the expected increase in food demand by 2050 will not be accompanied by the necessary increase in the relative rate of yield progress of major grain crops (wheat, maize, rice, and soybean), even considering breakthroughs as photosynthesis improvement *via* bioengineering (Hall and Richards, 2013). Additionally, grain yield (GY) losses of 8–43% respect to present-day yields are estimated for these crops, as climate change is predicted to increase temperatures and the frequency of extreme events such as drought (IPCC, 2014). This scenario will be accompanied by a largely augmented demand of water for direct human use, all trends that will require technologies aimed to improve and secure crop production with maximum resource-use efficiency and low environmental impact.

The difference between potential and actual GY varies extensively depending on the production area and the unpredictable climate. Water deficits of variable duration and intensity are among the main determinants of mentioned GY losses (Aramburu et al., 2015; Rattalino Edreira et al., 2018), and breeding efforts to increase crop tolerance to abiotic stress represent an environment-friendly avenue to reduce this gap.

Researchers worldwide have been working during decades applying different breeding strategies to increase crops GY under unfavorable environments (Campos et al., 2004), in a process that traditionally takes a long way from the initial stage in nurseries or *gene discovery* in labs to final adoption by farmers (Hall and Richards, 2013).

The present study contributes to a better understanding of the possibilities, difficulties and significant time requirements that occur when a transgenic technology developed in a model plant such as Arabidopsis is upgraded to evolutionary distant and economic important crops like wheat or soybean. A successful case is presented here: wheat and soybean transformed with the sunflower transcription factor gene *HaHB4* (*Helianthus annuus HomeoBox 4*). Transformed cultivars of both species are expected to be released to the market soon (probably during 2020), and this success was achieved through common and cooperative efforts of molecular biologists and agronomists from public institutions and private companies, who were able to overcome the additional obstacle usually imposed by epistemological barriers (Sadras and Richards, 2014).

## Although Genetically Modified Crops Have Been Adopted Worldwide, Second Generation is Absent in the Market

Since 1996, genetically modified (GM) crops have been adopted by farmers worldwide because they increase food and feed production efficiently by generating plants with higher GY in reasonably short times. The main advantage of transgenic technologies is the possibility to overcome sexual incompatibilities between plants and species barriers allowing the introduction of genes from unrelated organisms such as bacteria, fungi or other plants and also from viruses. Genetically modified organisms (GMOs), however, triggered controversies both in adopting and non-adopting countries, although stringent regulatory processes for food/feed and environmental safety were implemented and applied. Furthermore, several countries have established mandatory GMO labeling whereas voluntary labeling is preferred by others (Kamle et al., 2017). Most of the recently developed commercial GM crops exhibit herbicide tolerance, insect resistance or both traits stacked.

The second generation of GM crops was projected to mitigate abiotic stress effects. However, such crops are not commercially available so far and this is due to several reasons. Firstly, most evaluated events failed to translate benefits observed in controlled environments to field conditions (Passioura, 2012). Additionally, a long process is needed to release a GM product to the market, and this is due to regulation requirements mostly as a consequence of the bad public perception about GMOs (Blancke et al., 2015; Fernbach et al., 2019). Huge investments are needed to accomplish such requirements and have limited often attempts to advance GMOs at different stages of development.

An exception to the rule is the maize hybrid expressing a bacterial RNA chaperone that was released for use in a limited, drought-prone region of the United States (Castiglioni et al., 2008). Other drought-tolerant GMOs were developed but not released, such as sugarcane expressing a betaine gene and exhibiting augmented sugar content under water deficits (GM Approval Database, 2019).

## Upscaling Drought Tolerance From the Pot to the Crop

The advent of molecular genetics brought a pronounced increase in the number of studies involving plant transformation aimed to improve crop performance under water deficit conditions. The initial excitement was soon followed by the striking evidence of serious difficulties to scale up from individual plants grown in pots to communal plants in the field (Passioura, 2012). Lack of success has been usually linked to two weaknesses. One was the lack of a clear understanding of the benefits/drawbacks of a gene/trait at the crop rather than at the single plant level. For instance, compensations that usually take place when moving from plants to crops are of high importance (Pedró et al., 2012). Also, breeders and agronomists do not deal usually with growing conditions for which survival traits may represent an advantage (i.e. very low yielding environments). Traits of value are expected to represent an actual benefit in GY under stressful conditions with no penalty in the high yielding ones. The second weakness was poor knowledge of the variability in drought scenarios (i.e. opportunity, extent and intensity of drought) and their frequency (Chapman et al., 2000) in what breeders describe as the target population of environments (Cooper et al., 2005). Such variability usually receives little if any attention by molecular biologists (Passioura, 2006).

The water budget of an environment depends upon rainfall distribution and soil characteristics, which combined with evaporative demand, regulate the capacity of plants to hold maximum transpiration or reduce it (Sadras and Milroy, 1996). Reduced transpiration leads to the occurrence of water deficits of variable intensity and duration (Chapman et al., 2000; Connor et al., 2011). Rainfall distribution, together with temperature patterns set the limits to crop choice by farmers in rainfed systems, basically between the monsoon climate type (prevalence of summer crops) and the Mediterranean climate type (prevalence of winter crops). There are, however, humid and sub-humid areas where total rainfall allows year-round cropping systems but many times with large intra-seasonal variability if there is an occurrence of drought (Harrington and Tow, 2011). Simultaneously to climate, the soil type (i.e. texture) and its condition (e.g. compaction) affect total plant-available soil water storage as well as the amount that is readily available to plants (Passioura, 1991; Dardanelli et al., 2004).

Assuming cycle duration and its partitioning between vegetative and reproductive phases have been optimized to the water budget of each environment (Passioura, 2006), variable conditions experienced by the soil–canopy–atmosphere continuum along the cycle may pose an additional challenge when breeding crops for drought-prone regions. Passioura (2006) proposed to consider three main issues when evaluating a trait for these regions. The trait should increase (i) water use by transpiration (WUt, in mm) of the limited water supply, (ii) transpired water use efficiency for biomass production (WUEt: biomass produced per unit of water transpired, in kg ha^−1^ mm^−1^), and/or (iii) biomass allocation into harvestable products, namely harvest index in grain crops (HI: grain biomass/total biomass). These recommendations are based on our understanding of the physiological determination of GY at a crop level (Eq. 1)

(1) $$\text{GY}\;(\text{kg}\;{\text{ha}}^{-1})=\text{WUt}\times \text{WUEt}\times \text{HI}$$

Therefore, the ability of a gene/trait to cope with water constraints should be analyzed within the conceptual framework of Eq. 1 and the characteristics of the target environment (**Table 1**). The latter will settle whether a trait is favorable or unfavorable for a breeding program.

**Table 1:** Crop level analysis of the aptitude to cope with drought-prone environments of some traits.

Trait	Physiological GY Determinant	Target Environment
High ^12/13^C discrimination (C3 species)	WUEt	Terminal drought
Low ^12/13^C discrimination (C3 species)	WUEt	Mild, transient stress
Spike photosynthesis (wheat)	WUEt	All
Leaf rolling	WUEt	All
Increased boundary layer by wax or pubescence	WUEt	Terminal drought
Radiation use efficiency (light and N distribution within the canopy)	WUEt	All
High stomata sensitivity (i.e. fast stomata closure)	WUt	Terminal drought
Low stomata sensitivity	WUt	No terminal drought
Increased root growth	WUt	Deep water source
Osmotic adjustment	WUt	Transient stress, no terminal drought
Stay-green	WUt	No terminal drought
High floret fertility, reduced grain abortion	HI	Transient stress at flowering, no terminal drought
Pre-anthesis partitioning to stem carbohydrates	HI	Terminal drought

Adapted from Passioura (2006); Reynolds et al. (2007), Tardieu (2012) and Sadras and Richards (2014).

## The Story of HaHB4, From the Bench to the Field

It is well known and documented that there are plant species exhibiting a high tolerance to certain abiotic stress factors and others more susceptible to them (Boscaiu et al., 2012). Moreover, it is possible to find different varieties of the same species with differential tolerance to a stress factor for which traits conferring tolerance have been pyramided along centuries, first by farmers and subsequently by professional crop breeders. For example, there are rice lines tolerant to drought, extreme temperatures or salinity (https://www.irri.org/climate-change-ready-rice). Among crops, the sunflower is a species exhibiting broad adaptation (Debaeke et al., 2017), a characteristic for which responsible genes have been identified. Unfortunately, genetic tools as characterized mutants are not available and the complex sunflower genome was revealed only very recently (Badouin et al., 2017). Due to the complexity of molecular networks displayed when plants sense stressing conditions, transcription factors (TFs), as master switches, were good candidates to start the research.

TFs are proteins able to recognize and bind specific short DNA sequences present in their target regulatory regions; i.e. promoters, enhancers, introns, etc. These proteins are particularly abundant in the plant kingdom, representing about 6% of total proteins (Riechmann, 2002; Ribichich et al., 2014). Plant TFs have been classified in families, subfamilies, and subgroups especially according to their DNA binding domains. However, other features are also important for such classification including gene structure, the presence of other motifs and domains as well as their role in plant development. Many TF families are shared between animals and plants and among them, 19 are more expanded in the plant kingdom suggesting a frequent adaptive response, besides a higher genome duplication rate (Shiu et al., 2005).

Among TFs families, there is the superfamily of homeodomain (HD) containing proteins. The HD was defined as a conserved 60 aminoacid sequence that folds in three alpha helixes bound by a loop and a turn (reviewed in Viola and González, 2016). This domain was discovered (and named as HD) in mutants of *Drosophila melanogaster* exhibiting the ectopic expression or mutation of a HD encoding gene which caused a homeotic effect, i.e. the change of a body segment by another. Examples of these TFs are *Antennapedia* and *Bithorax* (Gehring et al., 1994). In plants, HD TFs have not been assigned homeotic functions, but they play many roles in development, hormone signaling and the response to environmental factors (Viola and González, 2016).

The HD superfamily is divided into several subfamilies and among the latter, there is the HD-Zip (homeodomain-leucine zipper) family which is subdivided into four groups, named I to IV, according to their different structures and roles. Even though HD and leucine zipper form part of TFs in other kingdoms, their association in a sole protein is exclusive of plants and this characteristic is shared by the four groups (Ariel et al., 2007). Among these four subfamilies, members of the so-called HD-Zip I subfamily have been associated initially with abiotic stress response (Ariel et al., 2007). Subsequently, several works [revised by (Perotti et al., 2017)] described the role of particular members in developmental events not necessarily associated with stress and also in biotic responses. There are 17 HD-Zip I members in the model plant Arabidopsis, a number that varies among species (Perotti et al., 2017). HD-Zip I TFs have been identified in all the species in which genomes have been sequenced; however, a small portion has been functionally characterized (Perotti et al., 2017). Phylogenetic trees resolved these proteins from different species in six clades (Arce et al., 2011). Coming back to the sunflower, it is noteworthy that this species has several divergent HD-Zip I TFs that cannot be clustered in the trees constructed with proteins from model species or crops (Arce et al., 2011). Among these divergent TFs, HaHB4 presents an abnormally short carboxy-terminal and a short size. Taking only its HD-Zip domain, the closest Arabidopsis members to HaHB4 are AtHB7 and AtHB12, which have been shown to participate as positive regulators in ABA-dependent drought and salinity responses (Olsson et al., 2004; Valdés et al., 2012; Ré et al., 2014). The overexpression of *AtHB7* conferred drought tolerance in Arabidopsis and tomato plants (Olsson et al., 2004; Mishra et al., 2012; Ré et al., 2014).

**Figure 1:**
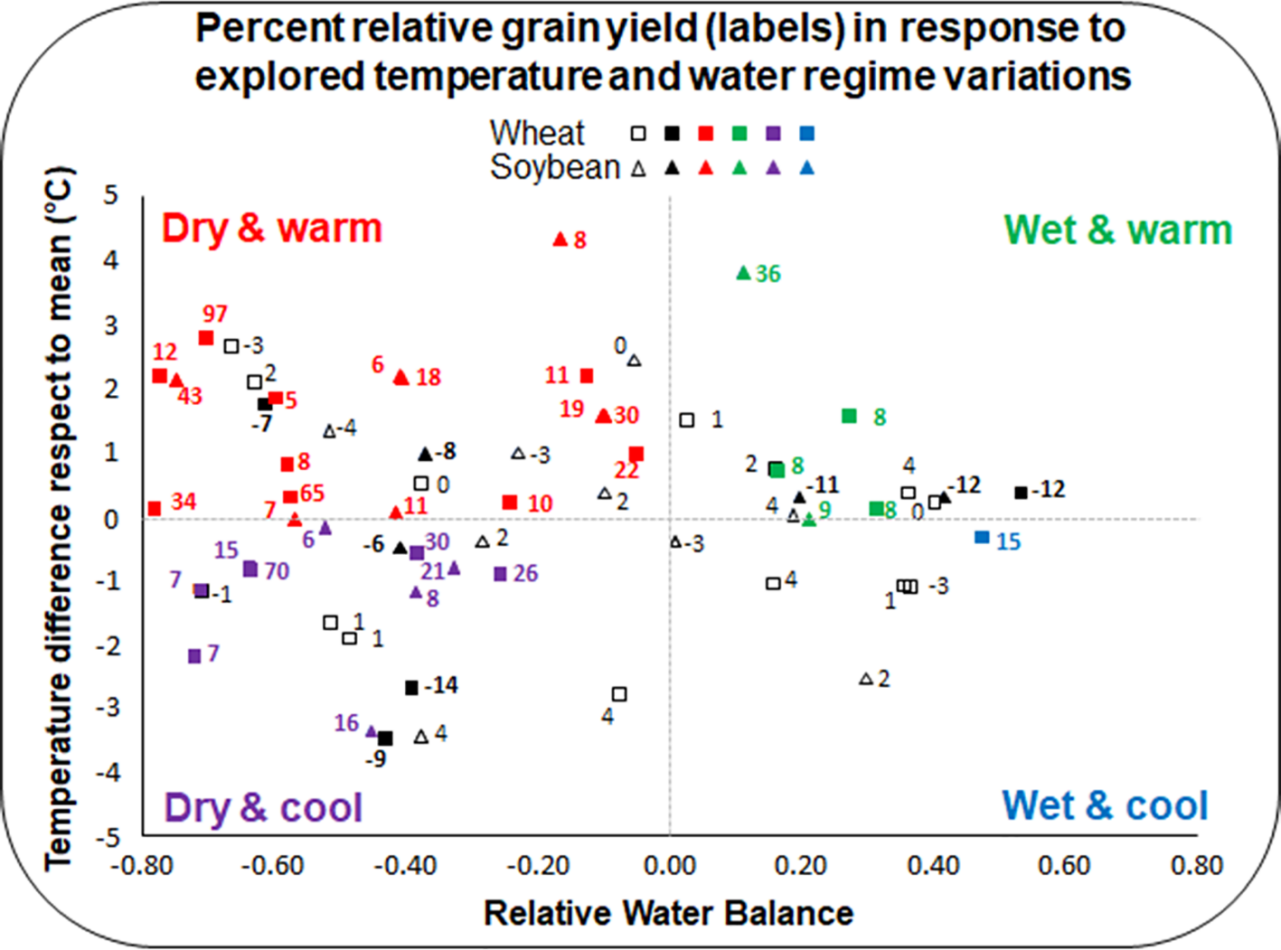
Relative grain yield response of transgenic wheat and soybean lines across environments. For each species (triangles for soybean and squares for wheat), symbols represent the combination of (i) the difference in mean temperature of each site respect to the mean across environments (*y* axis), and (ii) the relative water balance (RWB) of each site (*x* axis), being RWB=(Rainfall+Irrigation-PET)/PET (PET: potential evapotranspiration). Variation in relative grain yield (RGY) was computed as RGY = (GYtg-GYwt)/GYwt (GYtg: grain yield transgenic; GYwt: grain yield wild-type) and expressed in percent (values next to symbols). Different colors represent cases with (i) RGY ≥ 5% (GYtg > GYwt), in bolded non-black colors that identified the corresponding environmental group, (ii) RGY ≤ −5% (GYwt > GYtg), in bolded black, and (iii) −5% < RGY < 5% (GYtg = GYwt), in plain black.

At the beginning of the research reviewed here, and ignoring at that time the Arabidopsis genome as well as the functions of AtHB7 and AtHB12 and other members of the HD-Zip I family, the strategy to reveal the HaHB4 function was to study its binding specificity *in vitro* and its expression pattern in sunflower (Palena et al., 1999; Gago et al., 2002). The next step was to transform Arabidopsis plants overexpressing this TF (Dezar et al., 2005). Expression studies indicated that *HaHB4* is induced by water deficit and ABA (Gago et al., 2002). Arabidopsis transgenic plants, transformed with the sunflower TF, showed a drought-tolerant phenotype (Dezar et al., 2005). A deeper investigation about the mechanism triggered by this gene to confer drought tolerance indicated that it did not implicate stomata closure (which leads to drought tolerance but is usually accompanied by yield penalty in some environments) but a senescence delay *via* the inhibition of ethylene receptors (Manavella et al., 2006). Other signal transduction pathways are also regulated by this TF such as jasmonic acid enhancement, which leads to herbivory defense and inhibition of photosynthesis-related genes during darkness (Manavella et al., 2008a; Manavella et al., 2008b). However, the most important discovery was that Arabidopsis plants became, in certain form, ‘myopic’ to water deficit; they continue to grow when the stress was moderate and thus, the impact on productivity was reduced respect to control plants that exhibited stomata closure. In other words, when the plants were subjected to severe stress (not watered during 10-20 days), survival rates were much higher for GM plants expressing *HaHB4* than for wild-type controls (Dezar et al., 2005). Such a drought-tolerant phenotype was observed for many transgenic Arabidopsis plants expressing a variety of plant genes. However, the described trend did not hold when the same plants were grown under moderate water deficit (20–40% reduction of rosette area; Skirycz et al., 2011), a condition for which no clear trend was detected in yield penalty between GM and non-GM genotypes. By contrast, *HaHB4*-transgenics usually outyielded the wild-type controls across a wide range of field-tested growing conditions (**Figure 1**).

Described observations lead us to transform other species, particularly crops such as soybean and wheat, with constructs able to express *HaHB4* (González et al., 2019; Ribichich et al., 2020). Such GM crops expressing *HaHB4* outyielded their wild-type counterparts in a network of field trials that included a broad range of growing conditions, particularly in water balance and air temperature during critical reproductive stages (González et al., 2019; Ribichich et al., 2020). Yield data were supported by positive trends in its main physiological determinants (total biomass and biomass partitioning) as well as in floret fertility and grain numbers.

## Wheat and Soybean HaHB4: Crop Performance and Yield Improvement

Wheat and soybean expressing the cDNA corresponding to the sunflower *HaHB4* gene were tested in 37 and 27 field experiments, respectively (**Supplementary Table 1**; González et al., 2019; Ribichich et al., 2020)^1^. Genetic constructs used to transform crops shared *HaHB4* cDNA but not the promoters which were the UBI for wheat and the own *HaHB4* promoter for soybean. The parental wild-type cultivars (Cadenza for wheat and Williams 82 for soybean) together with the GM lines (IND-00412-7 for wheat and b10H for soybean) were sown in 13 or 14 sites during several years covering wide latitudinal (ca. 27°25’S to 39°50’S) and longitudinal (ca. 57°40’W to 65°28’W) ranges. Both crops experienced large differences in water balance as well as in mean and maximum temperatures (**Supplementary Table 1**). Considering all the range of tested environments (1,000–9,300 and 1,500–4,500 kg ha^−1^ average yield for wheat and soybean, respectively), the presence of *HaHB4* in the GM lines increased yield by 6% in wheat and by 4% in soybean, with no significant effect in crop phenology. Such a response is outstanding for commercial purposes because it allowed the incorporation of *HaHB4* to modern cultivars without altering the crop cycle, which has been already optimized to the target breeding area. When only the dry environments (i.e. negative water balance) were considered, the mean yield benefit from *HaHB4* increased to 16% in wheat and to 8.6% in soybean (**Figure 1**). Moreover, yield improvement was even larger (20% for wheat and 11% for soybean) when the dry environment was associated with warm mean temperatures (>20 and >22°C, respectively), while it remained important (12% and 5% for wheat and soybean, respectively) for the dry-cool condition (**Figure 1**). Differences in yield were always associated with grain number (GN) produced per unit area for both crops. This trend was partially compensated by a decrease in individual grain weight (GW) in soybean, whereas no clear trade-off effect was registered in this grain yield component for wheat. This contrasting response of GW to the increase in GN agrees with differences in the source-sink balance experienced by each species during grain filling (Borrás et al., 2004). Such balance recognizes specific (e.g. plasticity in the establishment of maximum seed volume; soybean > wheat) as well as environmental (e.g. irradiance offer during grain filling; wheat > soybean) constraints.

**Figure 2:**
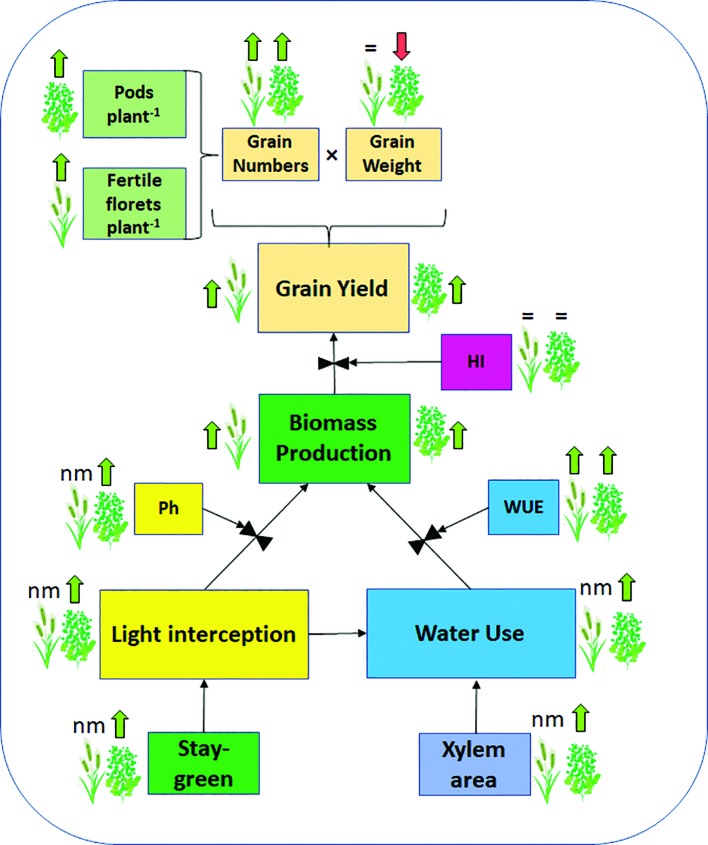
Schematic representation of HaHB4 effects. Effects of HaHB4 on some of the physiological determinants of wheat and soybean grain yield as well as on grain yield components (grain numbers and grain weight). Arrows indicate positive (green upward) or negative (red downward) effects respect to the wild-type when grown under water deficit. The equal sign indicates no change and nm indicates not measured. HI, harvest index; Ph, photosynthesis; WUE, water use efficiency.

**Figure 3:**
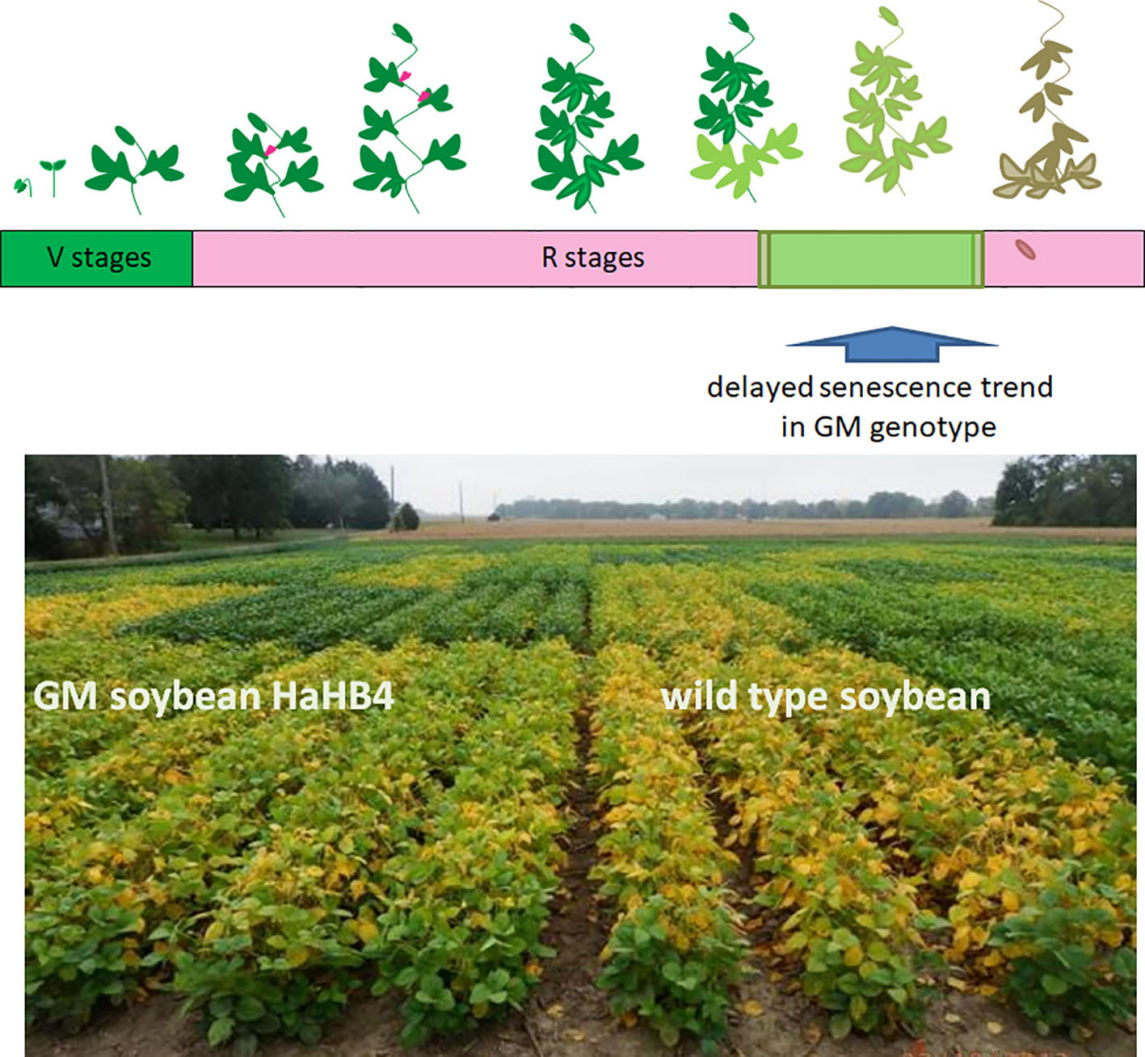
GM soybean exhibits delayed senescence compared with its wild-type control. Upper panel: schematic representation of the soybean life cycle. Lower panel: illustrative picture of one of the field trials performed comparing the wild type genotype (right) with the transgenic *HaHB4* one (left).

To improve our understanding of *HaHB4* effects on the ecophysiological determination of GY, detailed measurements were performed in controlled field experiments. The simplest method to study yield determination is to evaluate the total biomass produced along the crop cycle and the proportion of that biomass allocated to grains as proposed in Eq. 1 and summarized in **Figure 2**. In both crops, the expression of *HaHB4* caused an increase in total biomass with no change in harvest index (HI). As HaHB4 had no impact on crop phenology, the observed increase in total biomass could be attributed to increased crop growth rate, particularly during those periods that are critical for the determination of the main driver of grain yield (i.e. GN). This period spans between (i) the start of stem elongation on ca. 20 days before anthesis and grain set at the beginning of grain filling in wheat (Fischer, 1975; Fischer, 1985; Kirby, 1988), and (ii) pod formation and the beginning of grain filling in soybean (Board and Qiang, 1995; Jiang and Egli, 1995). The crop growth rate of wheat GM line exceeded that of the wild-type parental line by 68% during the critical period. This trend was in line with the increase registered in fertile florets per plant observed in GM lines, suggesting an improved floret survival (González et al., 2011). The fertile florets per spike and the number of spikes per plant constitute the fertile florets per plant, being the former similar (tiller spike) or higher (main stem spike), and the latter consistently higher, in the GM line compared to the wild type. For soybean, solar radiation interception and leaf photosynthesis were measured, both conducive to crop growth rate determination (Muchow et al., 1990). These two traits were higher for the GM line, the former during the entire critical period and the latter during grain filling. Improved light interception during the critical period resulted in more pods and branches per plant, whereas enhanced photosynthesis during grain filling was consistent with the clear visual observation of delayed senescence (**Figure 3**). Described stay-green improvement, which was also observed in model plant Arabidopsis (Manavella et al., 2006), probably prevented a complete trade-off between increased grain numbers and final individual grain weight, which can be expected when seed growth takes place under the sharp decrease in irradiance that usually occurs in autumn (Borrás et al., 2004).

When water availability is limiting growth and yield, water use (WU) and water use efficiency (WUE) are the main physiological determinants of crop performance (Eq. 1). In the case of wheat, the average WUE (estimated as the yield produced per unit of rainfall) of the 37 field experiments was 9.4% greater in the GM line than in the wild type, and WUE increased by 14.2% for environments with less than 300 mm rainfall. For soybean, detailed measurements of crop evapotranspiration along the cycle in plots exposed to contrasting water regimes (WW: well-watered; WD: water deficit) showed that the GM line used more water under both conditions, being the difference even higher under irrigation (17.3 and 27.2% increase in water used for WD and WW, respectively). The enhanced water use of the GM line could not be confirmed in a greenhouse experiment, where both cultivars had almost identical water use in pots held at contrasting water regimes (field capacity and 60% field capacity). Such a response is not surprising under the severe root confinement usually experienced in pot experiments, which do not allow for correct comparisons of this type of traits. Concurrently with mentioned differences in water use in the field, the hypocotyl diameter and xylem area were always larger in the GM line than in the wild-type soybean line, traits that have been associated with increased water conductivity and water use (Richards and Passioura, 1981). The fact that water use was reduced under water deficit, even in the GM line, suggests that some degree of stomata closure may have occurred, reducing water loss but with low impact in CO_2_ exchange (Liu et al., 2005). This response is in line with the increased WUE (≥22%) to produce biomass, and yield of the GM line when exposed to water deficit, and with the augmented photosynthetic rate observed in this germplasm during grain filling (commented in the previous paragraph). The results obtained in wheat and soybean crops are promising, showing that *HaHB4* may help to mitigate yield reductions in drought-prone environments.

## The Long Regulatory Process

**Figure 4:**
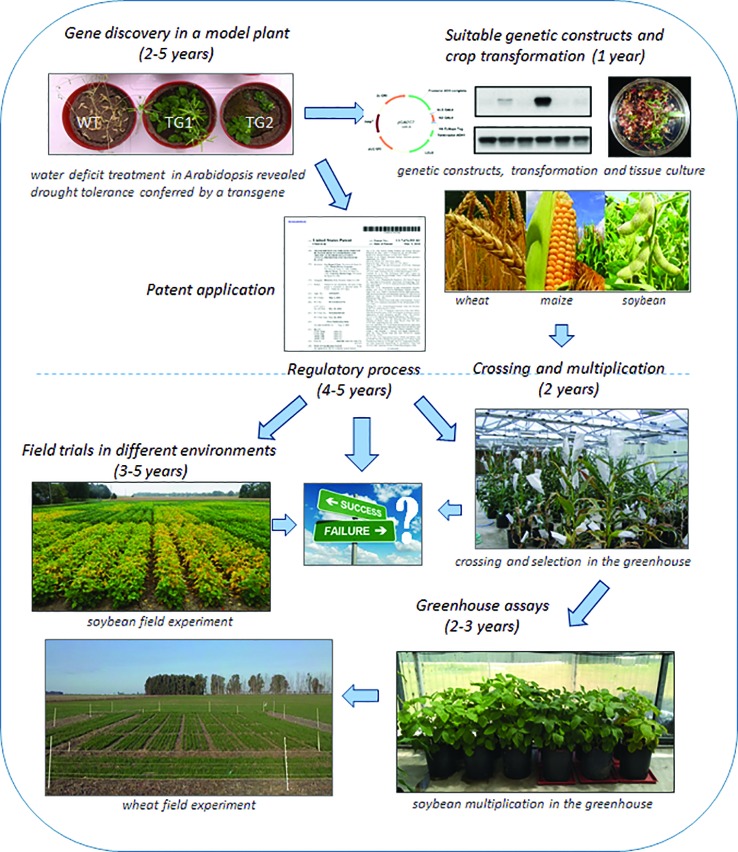
Research and development pipeline to upscale a technology from a model plant to a crop. Schematic representation of the whole process necessary to arrive from gene discovery (first step) to a genetically modified crop converted into a market product (last step).

Developing new technology is a long journey, from the hypothesis to its verification in the plant model, to the posterior projection into agronomic relevant crops and, when successful, the selection of the best candidate fulfilling the expected features (**Figure 4**). However, when all these stages are completed, another hard challenge starts. Since a precautionary approach is often favored for new technologies, a detailed safety assessment is required before their consumption or introduction into the environment.

While the approvals are obtained, these products are “regulated”, which means that they must be under strict control to guarantee no accidental release into the environment until its safety is proven. The regulatory stage is a mandatory process during which a lot of information covering different aspects of the new product must be delivered and presented to the respective authorities.

Focusing on a GM crop, the final objective of the regulatory phase is intended to establish that the new plant is like the conventional version except on the trait that was intentionally introduced by the genetic modification. The assumption under this *comparative approach* is that, if the conventional plant is considered secure, the genetically modified version would be equally safe. Behind this comparative approach that includes evaluation of different crop aspects (agronomic, reproductive, environmental, compositional, nutritional, etc.), there is a long and thorough process that involves measuring more than 80 parameters in which equivalence to the non-GM counterpart needs to be verified (Ayala et al., 2019; González et al., 2019).

Complementing the comparative approach, there is a set of distinctive (new) features that must be characterized. These include:

- Studying the new expression product. Any new molecule introduced into the crop by the genetic modification (usually a protein) must be deeply studied. In the case of a protein, its function, biochemical profile, putative allergenicity, and toxicity and digestibility, among other attributes, must be known.
- Examining if the new molecule and metabolites derived from it can interact with endogenous plant pathways.
- Describing the genetic material inserted, its origin, location within the genome, full characterization of the insertion, analyzing whether endogenous genes may have been interrupted by the genetic modification, analyzing putative production of unexpected products and its potential risk for consumption, and stability of the genetic modification.
- Many of the studies are planned in such a way that if any unintended effect is produced, it would be necessarily revealed by these studies, and that is why a wide agronomic and compositional characterization of the crop is a major part of any regulatory process (Ayala et al., 2019).

Field trials including the GMO cultivars are developed under different agronomic conditions. Simultaneously, the non-GM parental line and several commercial varieties are grown to control any effect of the genetic modification and provide a reference range of natural variability, respectively. A wide set of agronomic parameters that define the crop characteristics are measured during the life cycle and samples of different tissues are taken for further analysis.

Particular attention is taken to any parameter that may reveal a tendency of the GMO to become a weed or be more invasive than its wild-type counterpart. That is why reproductive physiology, persistence in the environment, sensitivity to stresses, diseases, and plagues are analyzed.

All described studies are completed by the developer since they involve the use of material protected by intellectual property rights and require a great investment of money, other resources and time. The results are presented to the regulatory authority of different countries and evaluated by experts in different areas. A post-submission period of communication between regulators and the developer usually follows, during which requests for additional information or even new studies may be required. The conclusions from these scientific analyses are then considered by governmental authorities, which take into consideration these evidence-based conclusions together with many other local interests.

Regulatory field trials are usually conducted in the main areas where the crop is cultivated. However, countries like China, Brazil, Japan, Colombia, Bolivia, South Africa, and the USA require local trials even when the transportability of the data has been proven (Garcia-Alonso et al., 2014).

Destinations, where a new GMO is presented for approval, include those where the crop will be cultivated and/or where its products will be shipped. Information presented for approval is usually that related to the use of the new GMO in the country of destination (information required for cultivation is different from that associated with consumption or use of imported products), although some countries/regions are extremely precautious and do not follow this rule.

The regulatory road is usually tortuous. Although several countries share the essence of a science-based safety evaluation process, there are still dissimilar requirements among regulatory authorities. Besides, there is a lack of mutual recognition on safety assessments that leads to redundant evaluations and asynchronous approvals that significantly impairs commodities commercialization (Stein and Rodríguez-Cerezo, 2010). In particular, some countries or regions are politically decisive players in this field. Specifically, the European Union embodies the stringency of a regulatory process requiring data not necessarily related to science or intention of use. Alternatively, China regulatory authorities run their own set of experiments to verify the data already generated by the developer.

One distinctive feature of the HB4^®^ technology (commercial name for HaHB4 introduced in different crops) is that the new expression product is a plant TF. Based on this singularity, this technology has faced additional scrutiny related to putative effects on non-target genes. Precedents on the extreme specificity of TF (an absolute requirement for the development of any individual), and evidenced on the different HaHB4-GM crops, do not support such speculation.

As a TF, the levels of *HaHB4* on the natural plant (sunflower) as well as in the GM crops expressing it are extremely low (at the nanogram per gram of dry weight; Alloatti et al., 2017). So low, that it can hardly be detected in the plant and, consequently, in its byproducts. If any concern could be raised by the expression of a foreign protein in a crop, in the case of HaHB4 it would be even lower than for other proteins.

Independently of the uniqueness of HaHB4, we have a familiarity with this protein, since sunflower has been in the animal and human diets for centuries. Besides, HaHB4 is similar to proteins already present in animals and plants, even working as TF.

Among the two HaHB4 crops close to reaching the market, HB4^®^ wheat has faced some baseless negative reactions. This is not unexpected considering the anti-GMO movements. However, it is sadly remarkable that those in charge of evaluating the (environmental/commercial) benefits or the safety of new technology, let this putative future public perception determine their decisions (Fernbach et al., 2019). This reaction is supported by the statement that this would be the first GM wheat. However, it is not true. There is a precedent for GM wheat, which completed the approval process in four different countries though it was never commercialized (GM Approval Database, 2019). Concerning this, another false statement is done: this event was rejected by the regulatory authorities. The truth is that the developers shelved the glyphosate-tolerant wheat in 2004 amid market concern about rejection from foreign buyers (Ingwersen, 2019). Besides these two countries where a withdrawal took place (the United States and Australia), this former GM wheat is approved for food and feed use in two other countries (New Zealand and Colombia, GM Approval Database, 2019).

This unique and interrupted intent to introduce GM wheat into the market sustain the feeling that this crop has been kept aside from the improvements that genetic modification may provide (Wulff and Dhugga, 2018; Asseng et al., 2019). Despite the public’s distaste for GM foods, especially in Europe, many genetically engineered products have already reached the market (GM Approval Database, 2019). In addition to public resistance to GM crops in general, wheat faces its particular challenges since twenty percent of humans’ calories and about the same percentage of protein intake come from wheat. In Argentina, 80% of the cultivated wheat is exported to Brazil. If buyers will not accept GM wheat, farmers will not grow it. According to a news release survey we conducted, based on articles selected through specific keywords, both in national and international media, stakeholders related to wheat exports speculate about potential market reactions if GM wheat is approved in Argentina, fearing a loss of customers based on not being able to deliver wheat that matches non-GM specifications. To get accurate, and credible information about identifying GM wheat, in the following section we perform a classification to distinguish between GM wheat and non-GM wheat, and between different “events” of GM wheat.

Another negative perception targeted GM wheat is that we are talking about human food and the direct consumption of a GMO. However, GM-derived products are already present in the food chain. For example, glyphosate-tolerant soybean MON-ØØ4Ø32-6 has been in the market for more than 20 years and soybean flour is regularly used as a supplement in several food products. Similarly, insect-resistant corn MON-ØØ81Ø-6 is widely cultivated and consumed since it was approved more than a couple of decades ago (GM Approval Database, 2019). Moreover, some of them are already consumed without prior processing (plum, cassava, apple). So, is there a real difference in approving this GMO or is it just a matter of perception?

Checking the history of GMOs, we can find that even in cases where there are no commercial interests behind a GM technology and its modification is attending a health issue as in the case of vitamin A-fortified golden rice (Dubock, 2014; Moghissi et al., 2018), lack of funding and anti-GMO movements can delay (or even prevent) a benefit to reach those who could be assisted by it.

## Transgenic Crops Can Be Differentiated On-Farm From the Non-Transgenic Ones by Spectral Analyses

Remote sensing techniques, such as spectrometry, are increasingly used for plant phenotyping. The spectrum of energy reflected by the plant is closely associated with absorption at certain wavelengths that are linked to specific characteristics or plant conditions. Spectrometers can acquire detailed information regarding the electromagnetic spectrum in a short time, making this technology ideal for assessing genotypes within a few hours. This would enable the estimation of multiple morpho-physiological and physicochemical traits, which would be otherwise impossible to evaluate due to the time and cost involved (Garriga et al., 2017).

For the estimation of plant traits, most previous studies have mainly resorted to the use of Vegetation Indices, while less attention was paid to the analysis of the full spectrum. The use of reflectance data and machine learning algorithms for plant phenotyping purposes using the full spectrum has been only recently addressed by the scientific community (Chlingaryan et al., 2018; El-Hendawy et al., 2019; Meacham-Hensold et al., 2019).

The capability of a spectrometer for the characterization of soybean, maize, and wheat in field experiments has been already explored by Rigalli et al. (2018). The objective of this work was to select different wavelengths intervals of the spectral reflectance curve (within the 632–1,125 nm range) as features for on-farm classification using machine learning methods. Two different classifications were presented, species selection and growth stage identification. An accuracy of 92% was reached for species classification, while 99% was obtained for stage classification. Besides, a new index was proposed that outperformed established vegetation indices under analysis, which showed the potential advantage of using this type of device. This fact indicated that a collection of field spectral data could be more representative of plant phenotyping than the information given by single vegetation indices.

**Figure 5:**
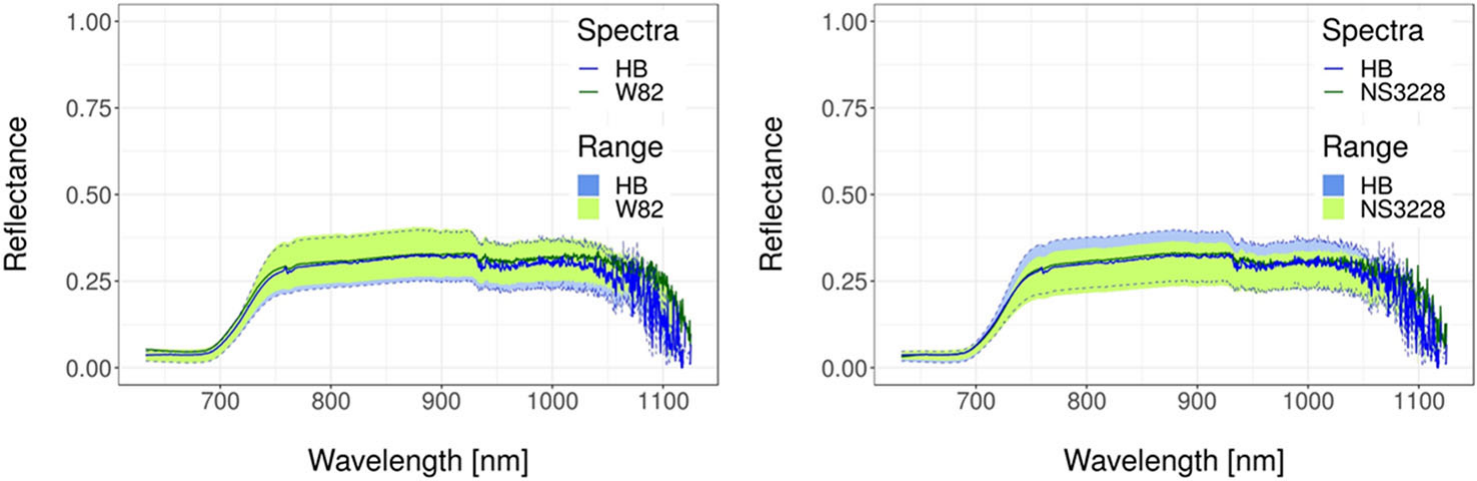
Spectral reflectance curve datasets for soybean. Transgenic versus wild-type and transgenic versus commercial. Solid lines represent typical spectral reflectance curves. Shaded regions represent the data range for each genotype. Green regions represent the data range of the non-transgenic genotypes. Blue regions with dashed border lines represent the dataset range of the transgenic genotypes. Spectra were obtained as described in **Supplementary File 1** whereas details of experiments are in **Supplementary Table 1** and in Ribichich et al. (2020).

**Figure 6:**
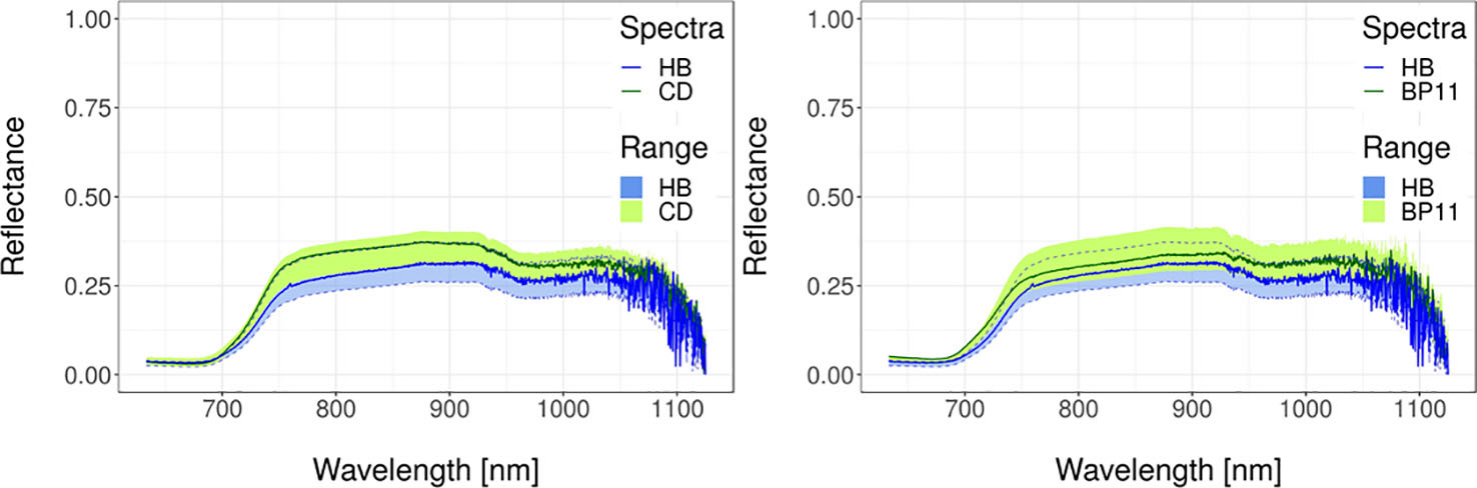
Spectral reflectance data for wheat. Transgenic versus wild-type (left) and transgenic versus commercial (right). Solid lines represent typical spectral reflectance curves. Shaded regions represent the data range for each genotype. Green regions represent the data range of the non-transgenic genotypes. Blue regions with dashed border lines represent the dataset range of the transgenic genotypes. Spectra were obtained as described in **Supplementary File 1** whereas details of experiments are in **Supplementary Table 1** and in González et al. (2019). Transgenic is cultivar IND-00412-7 (HB), wild-type is cultivar Cadenza (CD), and commercial cultivar is Baguette Premium 11 (BP11).

In this section, we present spectral analyses of field-grown wheat and soybean crops through field-collected full-spectrum data, in an attempt to differentiate GM genotypes from their wild type and commercial cultivars (**Figures 5** and **6**). Thirty spectral reflectance curves (ten per plot, with three replications) were collected for each genotype. Each dataset contained spectra from two genotypes, giving 60 spectra per dataset to feed the machine learning algorithm. Detail of methods (ANN: Artificial Neural Networks; SVM: Support Vector Machine; RF: Random Forest) can be found in the supplementary material (**Supplementary File 1**) whereas details of experiments are summarized in **Supplementary Table 1**.

### Transgenic Versus Wild Type Identification in Soybean and Wheat Genotypes

The best discrimination between GM and wild-type soybean cultivars was achieved with the ANN algorithm (**Table 2**), which reached values ≥70% in six out of 13 cases. Regarding the analyzed environments, the best performance was obtained for the water deficit condition (Environment II). Overall, the maximum standard deviation as a measure of uncertainty in the classification was 20%. In the case of wheat (**Table 3**), collected data belonged to a single environment and included two GM lines and the wild type at different growth stages. In this case, the precision of the classification increased when phenological differences between genotypes were reduced (bolded data in **Table 3**). The highest result for the GM/wild-type comparison (IND-00412-7(HB4)/Cadenza) was obtained on 130 days after sowing when both genotypes were at the boot stage. The outstanding precision value reached at this stage for wheat (96 ± 4% for ANN and 99 ± 8% for SVM) outperformed those registered for soybean (**Table 2**). This high precision allowed us to conclusively distinguish the presence of a GM wheat cultivar from its parental wild type through spectral analysis.

**Table 2:** Classification accuracy of three machine learning algorithms for discrimination between soybean genotypes.

	Environment I (Pergamino irrigated)					Environment II (Pergamino water deficit)					Environment III (IAL-Santa Fe)		
**DAS^a^**	**37**	45	**66**	87	100	37	**45**	**66**	**87**	100	**49**	**71**	**96**
**Growth Stage**	**V8**	R1	**R5**	R6	R7	V8	**R1**	**R5**	**R6**	R7	**R3**	**R5**	**R6**
**ANN**	0.69	0.65	**0.72**	0.57	0.55	0.68	**0.74**	**0.72**	**0.72**	0.58	**0.7**	0.52	**0.7**
**SVM**	0.66	0.64	**0.70**	0.57	0.54	0.67	**0.74**	**0.71**	0.65	0.56	0.68	0.52	**0.71**
**RF**	**0.71**	0.63	0.62	0.5	0.61	0.56	0.68	0.64	0.69	0.57	0.64	0.53	0.66

DAS, days after sowing; ANN, Artificial Neural Networks; SVM, Support Vector Machine; RF, Random Forest. Growth stages according to Fehr and Caviness (1977).

Tested soybean genotypes were b10H (GM) and W82 (wild-type parental). Results expressed as b10H/W82. Bolded data indicate results equal or higher than 70%.

**Table 3:** Classification accuracy of three machine learning algorithms for discrimination between wheat genotypes.

DAS Growth Stage (GM/Wild-type)	IND-00412-7*/CD			IND-1015*/CD		
	81	130	136	81	130	136
	1N-12/1N-15	BS+2/BS+2	Ant+2/Ant+1	1N-15/1N-15	Ant/BS+2	Ant+6/Ant+1
**ANN****^a^**	0.72	**0.96**	**0.91**	**0.82**	0.79	0.68
**SVM**	0.77	**0.99**	**0.94**	**0.78**	0.74	0.68
**RF**	0.71	**0.72**	**0.82**	**0.65**	0.67	0.54

a 1N, first node visible; Ant, anthesis; BS, boot stage. Other abbreviations as in **Table 2**.

Wheat genotypes used in this analysis were genotypes (i) IND-00412-7 (GM) and wild-type Cadenza (CD), and (ii) IND-1015 (GM) and CD. The growth stage is indicated as the nearer stage plus or minus calendar days from this stage. Bolded data indicate small variation between growth stages of the compared genotypes.

* IND-1015 come from the isogenic transgenic HaHB4 event obtained in Cadenza background (IND-00412-7), then introgressed in a pre-commercial advanced line.

### Identification of Transgenic Versus Commercial Genotypes in Soybean and Wheat

Using the same approach, transgenic (b10H, OECD nomenclature IND-00412-5) vs commercial (NS3228) soybean genotypes were evaluated across three environments (**Table 4**). According to these results, ANN obtained again the best performance, reaching values ≥70% in nine out of 13 cases. SVM also reached good results, showing values ≥70% in eight out of 13 cases. Classification accuracy across all environments was >70% in at least three developmental stages. Also, spectral reflectance data assessed at R5 (start of seed growth) showed classification accuracy values ≥79% for all analyzed environments. The performance improved under water deficit (Environment II) and high temperature (Environment III), reaching 82–86% and 84% (across methods), respectively. Standard deviations did not go beyond 16%.

**Table 4:** Classification accuracy of three machine learning algorithms for discrimination between transgenic and commercial soybean genotypes.

	Environment I (Pergamino irrigated)					Environment II (Pergamino water deficit)					Environment III (IAL-Santa Fe)		
**DAS**	**37**	45	**66**	87	100	37	**45**	**66**	**87**	100	**49**	**71**	**96**
**Growth Stage**	**V8**	R1	**R5**	R6	R7	V8	**R1**	**R5**	**R6**	R7	**R3**	**R5**	**R6**
**ANN****^a^**	**0.72**	0.67	**0.79**	**0.76**	0.56	**0.72**	0.68	**0.82**	**0.81**	0.6	**0.81**	**0.84**	**0.71**
**SVM**	**0.71**	0.64	**0.79**	**0.75**	0.56	0.68	0.69	**0.86**	**0.78**	0.59	**0.80**	**0.84**	**0.70**
**RF**	**0.72**	**0.70**	0.62	**0.70**	0.54	0.57	0.62	0.68	0.69	0.61	0.65	**0.73**	**0.77**

a Abbreviations as in **Table 2**.

Soybean genotypes tested in this analysis were HB (GM) and NS3228 (commercial variety). Results expressed as HB/NS3228 (bolded data indicate ≥0.70).

As for wheat varieties, we confirmed that a reduced phenological difference increases the discrimination capability between transgenic and non-transgenic cultivars. Accuracy of ANN was always highest (>80%), with standard deviations lower than 18%. Results for SVM were very similar to those obtained by ANN, and with standard deviations of similar magnitude (**Tables 5**).

**Table 5:** Classification accuracy of three machine learning algorithms for discrimination between transgenic and commercial wheat genotypes.

DAS	HB412/BP11			HB412/KP		
	81	130	136	81	130	136
**Growth Stage** (GM/Commercial)	**1N****^a^**-12/1N	**BS+2/Ant+11**	**Ant+1/Ant+17**	**1N-12/1N+2**	**BS+2/Ant+5**	**Ant+1/Ant+11**
**ANN**	0.89	0.84	0.81	0.86	0.93	0.99
**SVM**	0.82	0.73	0.9	0.87	0.91	0.99
**RF**	0.74	0.83	0.62	0.54	0.72	0.84

a Abbreviations as in **Tables 2** and **3**.

Tested wheat genotypes were HB412 (GM) and commercial varieties Baguette Premium 11 (BP11) and Klein Pegaso (KP). The growth stage is indicated as the nearer stage plus or minus calendar days from this stage. Bolded data indicate small variation between growth stages of the compared genotypes.

## Future Perspectives and Concluding Remarks

An estimated one-quarter of greenhouse gas emissions are associated with anthropogenic activities linked, directly or indirectly, to agriculture. At the same time, increases in frequency and intensity of extreme weather have adversely affected food security and ecosystems, contributing to desertification and land degradation in many regions (IPCC, 2014). While climate change will likely impact a crop´s yield and nutritional value, decreased agricultural outputs will fail to meet demands as population increases. Consequently, agriculture faces a major challenge: to enhance the resilience of global food systems and at the same time move towards carbon neutrality.

The unprecedented challenge of preserving our global environment today means we can no longer afford to increase agriculture production at the expense of environmental stability. This scenario leaves humanity with basically three avenues to reconcile agricultural productivity with environmental sustainability: reduce food waste, shift towards less meat intensive diets in the developed world, and use of the existing resources more sustainably.

Although second-generation GM crops have yet to reach global agriculture, they may contribute significantly to help us use existing resources more sustainably. An HaHB4 derived event for soybean has already been approved for cultivation in major agricultural territories like the United States, Brazil, and Argentina, with regulatory processes well advanced in other important geographies, such as China (developers’ public information). A similar wheat event is also being considered. This technology is expected to be in thousands of hectares during the 2019-2020 crop cycle, with the potential to be over one million hectares in two cycles after that, subject to farmers’ acceptance among other factors.

Drought tolerant wheat and soy crops, such as those described in this study, may yield more per unit of water used by plants. This resiliency may favor water-demanding double-cropping schemes that would otherwise be uneconomic. Sustainable intensification allowed by second-generation GM crops will result in improved carbon fixation, while less land is required to sustain current production outputs. These benefits may be of importance to a broader consumer audience, increasingly upset with our collective inability to preserve our terrestrial environment. Demonstrating these benefits at scale may generate an opportunity to re-signify GM perception derived from farmer-centric first-generation GMOs. In doing so, pressure could be mounted to streamline and synchronize global regulatory systems, making the process more affordable to a broader group of scientists and technology developers.

## Data Availability Statement

The datasets generated for this study are available on request to the corresponding authors.

## Author Contributions

NR, MR and MP performed spectral analyses. PM and FT wrote about the regulatory process. FG, KR and MO wrote about ecophysiological aspects whereas RC described HaHB4 story. RC and MO conceived and designed the manuscript.

## Funding

This work was supported by Agencia Nacional de Promoción Científica y Tecnológica, PICT 2015 2671.

## Conflict of Interest

PM and FT belong to INDEAR.

The remaining authors declare that the research was conducted in the absence of any commercial or financial relationships that could be construed as a potential conflict of interest.

## Footnotes

1 All this section is based on González et al., 2019 and Ribichich et al., 2020.

## References

[B1] Alloatti J. Blackford C. Burachik M. Chiozza M. Dezar C. Dicely I. (2017). Petition for determination of non-regulated status for the new plant variety HB4 soybean (IND-00410-5) intended for environmental release and food and feed use. (Riverdale, Madison, USA)

[B2] Aramburu F. Pablo J. Mercau J. L. Taboada M. Andrade F. H. Hall A. J. (2015). Field crops research potential for crop production increase in Argentina through closure of existing yield gaps. F. Crop Res. 184, 145–154. 10.1016/j.fcr.2015.10.001

[B3] Arce A. L. Raineri J. Capella M. Cabello J. V. Chan R. L. (2011). Uncharacterized conserved motifs outside the HD-Zip domain in HD-Zip subfamily I transcription factors; a potential source of functional diversity. BMC Plant Biol. 11, 42. 10.1186/1471-2229-11-42 21371298 PMC3060862

[B4] Ariel F. D. Manavella P. A. Dezar C. A. Chan R. L. (2007). The true story of the HD-Zip family. Trends Plant Sci. 12, 419–426. 10.1016/j.tplants.2007.08.003 17698401

[B5] Asseng S. Martre P. Maiorano A. Rötter R. P. O'Leary G. J. Fitzgerald G. J. (2019). Climate change impact and adaptation for wheat protein. Glob. Change Biol. 25, 155–173. 10.1111/gcb.14481 30549200

[B6] Ayala F. Fedrigo G. V. Burachik M. Miranda P. V. (2019). Compositional equivalence of event IND-ØØ412-7 to non-transgenic wheat. Transgenic Res. 28, 165–176. 10.1007/s11248-019-00111-y 30656492

[B7] Badouin H. Gouzy J. Grassa C. J. Murat F. Staton S. E. Cottret L. (2017). The sunflower genome provides insights into oil metabolism, flowering and Asterid evolution. Nature 546, 148–152. 10.1038/nature22380 28538728

[B8] Blancke S. Van Breusegem F. De Jaeger G. Braeckman J. Van Montagu M. (2015). Fatal attraction: the intuitive appeal of GMO opposition. Trends Plant Sci. 20, 414–418. 10.1016/j.tplants.2015.03.011 25868652

[B9] Board J. E. Qiang T. (1995). Assimilatory capacity effects on soybean yield components and pod number. Crop Sci. 35, 846–851. 10.2135/cropsci1995.0011183X003500030035x

[B10] Borrás L. Slafer G. A. Otegui M. E. (2004). Seed dry weight response to source-sink manipulations in wheat, maize and soybean: a quantitative reappraisal. F. Crop Res. 86, 131–146. 10.1016/j.fcr.2003.08.002

[B11] Boscaiu M. Donat P. Llinares J. Vicente O. (2012). Stress-tolerant wild plants: a source of knowledge and biotechnological tools for the genetic improvement of stress tolerance in crop plants. Not. Bot. Horti. Agrobo. 40, 323–327. 10.15835/nbha4028199

[B12] Campos H. Cooper M. Habben J. E. Edmeades G. O. Schussler J. R. (2004). Improving drought tolerance in maize: a view from industry. F. Crop Res. 90, 19–34. 10.1016/j.fcr.2004.07.003

[B13] Castiglioni P. Warner D. Bensen R. J. Anstrom D. C. Harrison J. Stoecker M. (2008). Bacterial RNA chaperones confer abiotic stress tolerance in plants and improved grain yield in maize under water-limited conditions. Plant Physiol. 147, 446–455. 10.1104/pp.108.118828 18524876 PMC2409020

[B14] Chapman S. C. Cooper M. Hammer G. L. Butler D. G. (2000). Genotype by environment interactions affecting grain sorghum. II. Frequencies of different seasonal patterns of drought stress are related to location effects on hybrid yields. Aust. J. Agric. Res. 51, 209. 10.1071/AR99021

[B15] Chlingaryan A. Sukkarieh S. Whelan B. (2018). Machine learning approaches for crop yield prediction and nitrogen status estimation in precision agriculture: a review. Comput. Electron. Agr. 151, 61–69. 10.1016/j.compag.2018.05.012

[B16] Connor D. Loomis R. Cassman K. (2011). “Photosynthesis,” in Crop Ecology: Productivity and Management in Agricultural Systems (Cambridge: Cambridge University Press), 262–291. 10.1017/CBO9780511974199.014

[B17] Cooper M. Podlich D. W. Smith O. S. (2005). Gene-to-phenotype models and complex trait genetics. Aust. J. Agric. Res. 56, 895–918. 10.1071/AR05154

[B18] Dardanelli J. L. Ritchie J. T. Calmon M. Andriani J. M. Collino D. J. (2004). An empirical model for root water uptake. F. Crop Res. 87, 59–71. 10.1016/j.fcr.2003.09.008

[B19] Debaeke P. Casadebaigm P. Flenetm F. Langlade N. (2017). Sunflower crop and climate change: vulnerability, adaptation, and mitigation potential from case-studies in Europe. OCL 24 (1), D102. 10.1051/ocl/2016052

[B20] Dezar C. A. Gago G. M. Gonzalez D. H. Chan R. L. (2005). Hahb-4, a sunflower homeobox-leucine zipper gene, is a developmental regulator and confers drought tolerance to Arabidopsis thaliana plants. Transgenic Res. 14, 429–440. 10.1007/s11248-005-5076-0 16201409

[B21] Dubock A. (2014). The politics of Golden Rice. GM Crops Food 5, 210–222. 10.4161/21645698.2014.967570 25437240 PMC5033200

[B22] El-Hendawy S. E. Al-Suhaibani N. A. Hassan W. M. Dewir Y. H. Elsayed S. Al-Ashkar I. (2019). Evaluation of wavelengths and spectral reflectance indices for high-throughput assessment of growth, water relations and ion contents of wheat irrigated with saline water. Agric. Water Manage. 212, 358–377. 10.1016/j.agwat.2018.09.009

[B23] Fehr W. R. Caviness C. E. (1977). Stages of soybean development. Special Report (Iowa, USA: Iowa State University), 87.

[B24] Fernbach P. M. Light N. Scott S. E. Inbar Y. Rozin P. (2019). Extreme opponents of genetically modified foods know the least but think they know the most. Nat. Hum. Behav. 3, 251–256. 10.1038/s41562-018-0520-3 30953007

[B25] Fischer R. A. (1975). Yield potential in a dwarf spring wheat and the effect of shading. Crop Sci. 5, 607–614. 10.2135/cropsci1975.0011183X001500050002x

[B26] Fischer R. A. (1985). Number of kernels in wheat crops and the influence of solar radiation and temperature. J. Agric. Sci. Cambridge 105, 447–461. 10.1017/S0021859600056495

[B27] Gago G. M. Almoguera C. Jordano J. Gonzalez D. H. Chan R. L. (2002). Hahb-4, a homeobox-leucine zipper gene potentially involved in abscisic acid-dependent responses to water stress in sunflower. Plant Cell Environ. 25, 633–640. 10.1046/j.1365-3040.2002.00853.x

[B28] Garcia-Alonso M. Hendley P. Bigler F. Mayeregger E. Parker R. Rubinstein C. (2014). Transportability of confined field trial data for environmental risk assessment of genetically engineered plants: a conceptual framework. Transgenic Res. 23, 1025–1041. 10.1007/s11248-014-9785-0 24733670 PMC4204004

[B29] Garriga M. Romero-Bravo S. Estrada F. Escobar A. Matus I. A. del Pozo A. (2017). Assessing wheat traits by spectral reflectance: do we really need to focus on predicted trait-values or directly identify the elite genotypes group? Front. Plant Sci. 8, 280. 10.3389/fpls.2017.00280 28337210 PMC5343032

[B30] Gehring W. J. Affolter M. Bürglin T. R. (1994). Homeodomain proteins. Annu. Rev. Biochem. 63, 487–526. 10.1146/annurev.bi.63.070194.002415 7979246

[B31] GM Approval Database (2019). http://www.isaaa.org/gmapprovaldatabase/.

[B32] González F. Capella M. Ribichich K. Curín F. Giacomelli J. Ayala F. (2019). Wheat transgenic plants expressing the sunflower gene HaHB4 significantly outyielded their controls in field trials. J. Exp. Bot. 70, 1669–1681. 10.1093/jxb/erz037 30726944 PMC6411379

[B33] González F. G. Miralles D. J. Slafer G. A. (2011). Wheat floret survival as related to pre-anthesis spike growth. J. Exp. Bot. 62, 4889–4901. 10.1093/jxb/err182 21705386

[B34] Hall A. J. Richards R. A. (2013). Prognosis for genetic improvement of yield potential and water-limited yield of major grain crops. F. Crop Res. 143, 18–33. 10.1016/j.fcr.2012.05.014

[B35] Harrington L. Tow P. (2011). “Principles of a systems approach to agriculture: some definitions and concepts,” in Rainfed Farming Systems. Eds. Tow P. Cooper I. Partridge I. Birch C., 45–74. 10.1007/978-1-4020-9132-2

[B36] Ingwersen J. (2019). USDA investigates unapproved GMO wheat found in Washington state.

[B37] IPCC (2014). Climate Change 2014: Synthesis Report. Contribution of Working Groups I, II and III to the Fifth Assessment Report of the Intergovernmental Panel on Climate Change. Eds. Core Writing Team, R. K. Pachauri, and L. A. Meyer. (Geneva, Switzerland: IPCC), 151 pp.

[B38] Jiang H. Egli D. B. (1995). Soybean seed number and crop growth rate during flowering. Agron. J. 87, 264–267. 10.2134/agronj1995.00021962008700020020x

[B39] Kamle M. Kumar P. Patra J. K. Bajpai V. K. (2017). Current perspectives on genetically modified crops and detection methods. Biotech 7, 219. 10.1007/s13205-017-0809-3 PMC5495694 28674844

[B40] Kirby E. J. M. (1988). Analysis of leaf, stem and ear growth in wheat from terminal spikelet stage to anthesis. Field Crops Res. 18, 127–140. 10.1016/0378-4290(88)90004-4

[B41] Liu F. Andersen M. N. Jacobsen S. E. Jensen C. R. (2005). Stomatal control and water use efficiency of soybean (Glycine max L. Merr.) during progressive soil drying. Environ. Exp. Bot. 54, 33–40. 10.1016/j.envexpbot.2004.05.002

[B42] Manavella P. A. Arce A. L. Dezar C. A. Bitton F. Renou J. P. Crespi M. (2006). Cross-talk between ethylene and drought signalling pathways is mediated by the sunflower Hahb-4 transcription factor. Plant J. 48, 125–137. 10.1111/j.1365-313X.2006.02865.x 16972869

[B43] Manavella P. A. Dezar C. A. Bonaventure G. Baldwin I. T. Chan R. L. (2008a). HAHB4, a sunflower HD-Zip protein, integrates signals from the jasmonic acid and ethylene pathways during wounding and biotic stress responses. Plant J. 56, 376–388. 10.1111/j.1365-313X.2008.03604.x 18643970

[B44] Manavella P. A. Dezar C. A. Ariel F. D. Drincovich M. F. Chan R. L. (2008b). The sunflower HD-Zip transcription factor HAHB4 is up-regulated in darkness, reducing the transcription of photosynthesis-related genes. J. Exp. Bot. 59, 3143–3155. 10.1093/jxb/ern170 18603614

[B45] Meacham-Hensold K. Montes C. M. Wu J. Guan K. Fu P. Ainsworth E. A. (2019). High-throughput field phenotyping using hyperspectral reflectance and partial least squares regression (PLSR) reveals genetic modifications to photosynthetic capacity. Remote Sens. Environ. 231, 111176. 10.1016/j.rse.2019.04.029 31534277 PMC6737918

[B46] Mishra K. B. Iannacone R. Petrozza A. Mishra A. Armentano N. La Vecchia G. (2012). Engineered drought tolerance in tomato plants is reflected in chlorophyll fluorescence emission. Plant Sci. 182, 79–86. 10.1016/j.plantsci.2011.03.022 22118618

[B47] Moghissi A. A. Jaeger L. M. Shafei D. Bloom L. L. (2018). Regulatory science requirements of labeling of genetically modified food. Crit. Rev. Biotechnol. 38, 386–393. 10.1080/07388551.2017.1356804 29041813

[B48] Muchow R. C. Sinclair T. R. Bennett J. M. (1990). Temperature and solar radiation effects on potential maize yield across locations. Agron. J. 82, 338. 10.2134/agronj1990.00021962008200020033x

[B49] Olsson A. S. Engström P. Soderman E. (2004). The homeobox genes Athb12 and Athb7 encode potential regulators of growth in response to water deficit in Arabidopsis. Plant Mol. Biol. 55, 663–677. 10.1007/s11103-004-1581-4 15604708

[B50] Palena C. M. González D. H. Chan R. L. (1999). A monomer-dimer equilibrium modulates the interaction of the sunflower homeodomain leucine-zipper protein Hahb-4 with DNA. Biochem. J. 341, 81–87. 10377247 PMC1220332

[B51] Passioura J. B. (1991). Soil structure and plant growth. Aust. J. Soil Res. 29, 717–728. 10.1071/SR9910717

[B52] Passioura J. (2006). Increasing crop productivity when water is scarce—from breeding to field management. Agric. Water Manage. 80, 176–196. 10.1016/j.agwat.2005.07.012

[B53] Passioura J. B. (2012). Phenotyping for drought tolerance in grain crops. Funct. Integr. Genomics 39, 851–859. 10.1071/FP12079 32480835

[B54] Pedró A. Savin R. Slafer G. A. (2012). Crop productivity as related to single-plant traits at key phenological stages in durum wheat. F. Crop Res. 138, 42–51. 10.1016/j.fcr.2012.09.016

[B55] Perotti M. F. Ribone P. A. Chan R. L. (2017). Plant transcription factors from the Homeodomain-Leucine Zipper family I. Role in development and stress responses. IUBMB Life 69, 280–289. 10.1002/iub.1619.R 28337836

[B56] Ré D. A. Capella M. Bonaventure G. Chan R. L. (2014). Arabidopsis AtHB7 and AtHB12 evolved divergently to fine tune processes associated with growth and responses to water stress. BMC Plant Biol. 14, 150. 10.1186/1471-2229-14-150 24884528 PMC4064807

[B57] Rattalino Edreira J. I. Guilpart N. Sadras V. Cassman K. G. van Ittersum M. K. Schils R. L. M. (2018). Water productivity of rainfed maize and wheat: a local to global perspective. Agric. For. Meteorol. 259, 364–373. 10.1016/j.agrformet.2018.05.019 30224833 PMC6018065

[B58] Reynolds M. P. Saint Pierre C. Saad A. S. I. Vargas M. Condon A. G. (2007). Evaluating potential genetic gains in wheat associated with stress-adaptive trait expression in elite genetic resources under drought and heat stress. Crop Sci. 47 (S3), S172–S189. 10.2135/cropsci2007.10.0022IPBS

[B59] Ribichich K. F. Arce A. L. Chan R. L. (2014). “Coping with drought and salinity stresses: role of transcription factors in crop improvement,” in Climate Change and Plant Abiotic Stress Tolerance. Eds. Tuteja N. Gill S. S. (Weinheim, Germany: Wiley-VCH Verlag GmbH & CO KGaA), 641–684.

[B60] Ribichich K. F. Chiozza M. Ávalos-Britez S. Cabello J. V. Arce A. L. Watson G. (2020). Successful field performance in dry-warm environments of soybean expressing the sunflower transcription factor HaHB4. J. Exp. Bot. 10.1093/jxb/eraa064 PMC7260725 32140724

[B61] Richards R. A. Passioura J. B. (1981). Seminal root morphology and water use of wheat II. Genet. Var. Crop Sci. 21, 253–255. 10.2135/cropsci1981.0011183X002100020012x

[B62] Riechmann J. L. (2002). Transcriptional Regulation: A Genomic Overview. Arabidopsis Book Vol. 1 (USA: American Society of Plant Biologists), e0085. 10.1199/tab.0085 22303220 PMC3243377

[B63] Rigalli N. F. Montero Bulacio E. Romagnoli M. Terissi L. Portapila M. (2018). Identification and characterization of crops through the analysis of spectral data with machine learning algorithms, Argentinian Conference of Information Technology, M CAI-50 ISSN: 2525-0949. http://47jaiio.sadio.org.ar/sites/default/files/CAI-50.pdf.

[B64] Sadras V. O. Milroy S. P. (1996). Soil-water thresholds for the responses of leaf expansion and gas exchange: a review. F. Crop Res. 47, 253–266. 10.1016/0378-4290(96)00014-7

[B65] Sadras V. O. Richards R. A. (2014). Improvement of crop yield in dry environments: Benchmarks, levels of organisation and the role of nitrogen. J. Exp. Bot. 65, 1981–1995. 10.1093/jxb/eru061 24638898

[B66] Shiu S.-H. Shih M.-C. Li W.-H. (2005). Transcription factor families have much higher expansion rates in plants than in animals. Plant Physiol. 139, 18–26. 10.1104/pp.105.065110 16166257 PMC1203354

[B67] Skirycz A. Vandenbroucke K. Clauw P. Maleux K. De Meyer B. Dhondt S. (2011). Survival and growth of Arabidopsis plants given limited water are not equal. Nat. Biotechnol. 29, 212–214. 10.1038/nbt.1800 21390020

[B68] Stein A. J. Rodríguez-Cerezo E. (2010). International trade and the global pipeline of new GM crops. Nat. Biotechnol. 28, 23–25. 10.1093/jxb/err269 20062032

[B69] Tardieu F. (2012). Any trait or trait-related allele can confer drought tolerance: just design the right drought scenario. J. Exp. Bot. 63 (1), 25–31. 10.1093/jxb/err269 21963615

[B70] Valdés A. Övernäs E. Johansson H. Rada-Iglesias A. Engström P. (2012). The homeodomain-leucine zipper (HD-Zip) class I transcription factors ATHB7 and ATHB12 modulate abscisic acid signalling by regulating protein phosphatase 2C and abscisic acid receptor gene activities. Plant Mol. Biol. 80, 405–418. 10.1007/s11103-012-9956-4 22968620

[B71] Viola I. L. González D. H. (2016). “Structure and Evolution of Plant Homeobox Genes,” in Plant Transcription Factors. Ed. González D. H. (Amsterdam, Netherlands: Elsevier). Chapter 6.

[B72] Wulff B. B. H. Dhugga K. S. (2018). Wheat-the cereal abandoned by GM. Science 361, 451–452. 10.1126/science.aat5119 30072526

