# Supplementary material for: An Interdisciplinary Approach to Study the Performance of Second-generation Genetically Modified Crops in Field Trials: A Case Study With Soybean and Wheat Carrying the Sunflower HaHB4 Transcription Factor
Source: Front Plant Sci. 2020 Mar 6;11:178. doi: 10.3389/fpls.2020.00178 (PMC7069416; doi:10.3389/fpls.2020.00178)
Supplement: Supplementary file 1 [file DataSheet_1.docx]

**An interdisciplinary approach to study the performance of second-generation genetically modified crops in field trials: a case study with soybean and wheat carrying the sunflower HaBH4 transcription factor**

Fernanda Gabriela González^a^, Nicolás Rigalli^b^, Patricia Vivian Miranda^cg^, Martín Romagnoli^b^, Karina Fabiana Ribichich^d^, Federico Trucco^c^, Margarita Portapila^b^, María Elena Otegui^f*^, Raquel Lía Chan ^d*^

**Method for spectral analyses**

Canopy Spectral Reflectance (CSR) of each plot was measured using a STS-NIR spectrometer (Ocean Optics, Inc., Dunedin, FL). The instrument was sensitive to 1024 wavelengths in the range from 632nm to 1125nm with an optical resolution of 3nm. The upwelling light reflected from a 50 cm x 50 cm white reference material (WRM with 99% reflectance) was measured prior to each data acquisition allowing measurement during variable sky conditions. All measurements were carried with the sensor positioned at a nadir view 50 cm above the surface, thus the diameter of the measured footprint was approximately 26 cm. The integration time was adjusted in order to avoid saturation of the WRM signal. The electrical noise was measured through the occlusion of the spectrometer’s entrance slit and then subtracted from WRM and plot canopy measurements. Ten CSR measurements per plot were recorded. They were homogeneously distributed over the plot avoiding border effects.

In this work R software was used for processing data and applying artificial intelligence techniques (R Core Team, 2014). Three machine learning algorithms (MLA), Artificial Neural Networks (ANN, hidden layer of 4 neurons and weight decay factor of 1.10e-3), Support Vector Machine with Linear Kernel (SVM, cost value of 5) and Random Forests (RF, 500 decision trees) were trained for classification (Hassoun, 1995; Cortes and Vapnik, 1995; Breiman, 2001). Average classification accuracy for 10xCV was the metric chosen to test model performance.

**References**

Breiman, L. (2001) Random forests. *Machine Learning* 45,(1) 5-32. doi:10.1023/A:1010933404324

Core Team (2014) R: A Language and Environment for Statistical Computing. Vienna, Austria.

Cortes, C., and Vapnik, V. (1995). Support Vector Networks. *Machine Learning* 20, 273-297. doi: 10.1023/A:1022627411411

Hassoun, M. H. (1995) Fundamentals of Artificial Neural Networks. MIT Press.
