# Supplementary material for: An Interdisciplinary Approach to Study the Performance of Second-generation Genetically Modified Crops in Field Trials: A Case Study With Soybean and Wheat Carrying the Sunflower HaHB4 Transcription Factor
Source: Front Plant Sci. 2020 Mar 6;11:178. doi: 10.3389/fpls.2020.00178 (PMC7069416; doi:10.3389/fpls.2020.00178)
Supplement: Supplementary file 2 [file Table_1.docx]

**An interdisciplinary approach to study the performance of second-generation genetically modified crops in field trials: a case study with soybean and wheat carrying the sunflower HaBH4 transcription factor**

Fernanda Gabriela González^a^, Nicolás Rigalli^b^, Patricia Vivian Miranda^cg^, Martín Romagnoli^b^, Karina Fabiana Ribichich^d^, Federico Trucco^c^, Margarita Portapila^b^, María Elena Otegui^f*^, Raquel Lía Chan ^d*^

**Supplementary Table 1**

Description of experiments preformed. The environmental variables are those explored for critical period and grain filling (ca. -20 days prior to anthesis to yelow peduncle in wheat and from R3 to R7 in soybean) and and yield. Tmax: maximum average temperature, T mean: mean average temperature. Water balance= rain + irrigation - potential evapotranspiration. a and b denote early and late sowing dates. All experiments were rain-fed except when indicated by c: irrigated or d: water deficit by rain-out shelter. WT: wild type, GM: genetically modified. e and f indicate different fertilization rates. Table modified from González *et al*., 2019 and Ribichich *et al*., 2020.

**References**

González, F., Capella, M., Ribichich, K., Curín, F., Giacomelli, J., Ayala, F., et al. (2019). Wheat transgenic plants expressing the sunflower gene HaHB4 significantly outyielded their controls in field trials*. J. Exp. Bot.* 70, 1669-1681. doi:10.1093/jxb/erz037.

Ribichich, K.F., Chiozza, M., Ávalos-Britez, S., Cabello, J.V., Arce, A.L, Watson, G., Arias, C., Portapila, M., Trucco, F., Otegui, M.E., and Chan, R.L. (2019). Successful field performance in dry-warm environments of soybean expressing the sunflower transcription factor HaHB4. *J. Exp. Bot (in press);*  doi: 10.1093/jxb/eraa064
